# Antimetabolic Effects of Polyphenols in Breast Cancer Cells: Focus on Glucose Uptake and Metabolism

**DOI:** 10.3389/fnut.2018.00025

**Published:** 2018-04-16

**Authors:** Elisa Keating, Fátima Martel

**Affiliations:** ^1^Department of Biomedicine, Unit of Biochemistry, Faculty of Medicine, University of Porto, Porto, Portugal; ^2^CINTESIS, Center for Research in Health Technologies and Information Systems, University of Porto, Porto, Portugal; ^3^Instituto de Investigação e Inovação em Saúde, Universidade do Porto, Porto, Portugal

**Keywords:** polyphenols, Warburg effect, glucose uptake, glycolysis, breast cancer

## Abstract

In the last years, metabolic reprogramming became a new key hallmark of tumor cells. One of its components is a deviant energetic metabolism, known as Warburg effect—an *aerobic lactatogenesis—*characterized by elevated rates of glucose uptake and consumption with high-lactate production even in the presence of oxygen. Because many cancer cells display a greater sensitivity to glucose deprivation-induced cytotoxicity than normal cells, inhibitors of glucose cellular uptake (facilitative glucose transporter 1 inhibitors) and oxidative metabolism (glycolysis inhibitors) are potential therapeutic targets in cancer treatment. Polyphenols, abundantly contained in fruits and vegetables, are dietary components with an established protective role against cancer. Several molecular mechanisms are involved in the anticancer effect of polyphenols, including effects on apoptosis, cell cycle regulation, plasma membrane receptors, signaling pathways, and epigenetic mechanisms. Additionally, inhibition of glucose cellular uptake and metabolism in cancer cell lines has been described for several polyphenols, and this effect was shown to be associated with their anticarcinogenic effect. This work will review data showing an antimetabolic effect of polyphenols and its involvement in the chemopreventive/chemotherapeutic potential of these dietary compounds, in relation to breast cancer.

## Introduction

Breast cancer is the second most common cancer worldwide, after lung cancer, and it is the most common cancer among women (1). This type of cancer is the fifth cause of death from cancers in both sexes and the first in women (1). Nevertheless, 5-year survival rates in breast cancer patients are very high in high-income countries, reaching 80% and decreasing to 40% in low-income countries, mainly because of reduced availability of screening strategies (1, 2). The widespread adoption of screening mammography together with the use of post-menopausal replacement hormonal therapy accounts for the increased incidence of breast cancer observed since the 1970s (3), meaning that this disease is detected earlier and is now more preventable than ever.

Despite the overall promising statistics regarding breast cancer, global efforts must be undertaken in order to eradicate breast cancer as a chronic disease and to reduce mortality, treatment-associated morbidities and, importantly, the overwhelming emotional and economic burden associated with this disease. The Lancet Oncology Commission has very recently published a report listing cancer research priorities for the near future (4). Cancer research, management, and drug discovery during the last decades focused particularly on the discovery of new molecular targets and in the refinement of lead compounds, and these research areas are still part of that list of priorities in the United States (4).

**Figure 1:**
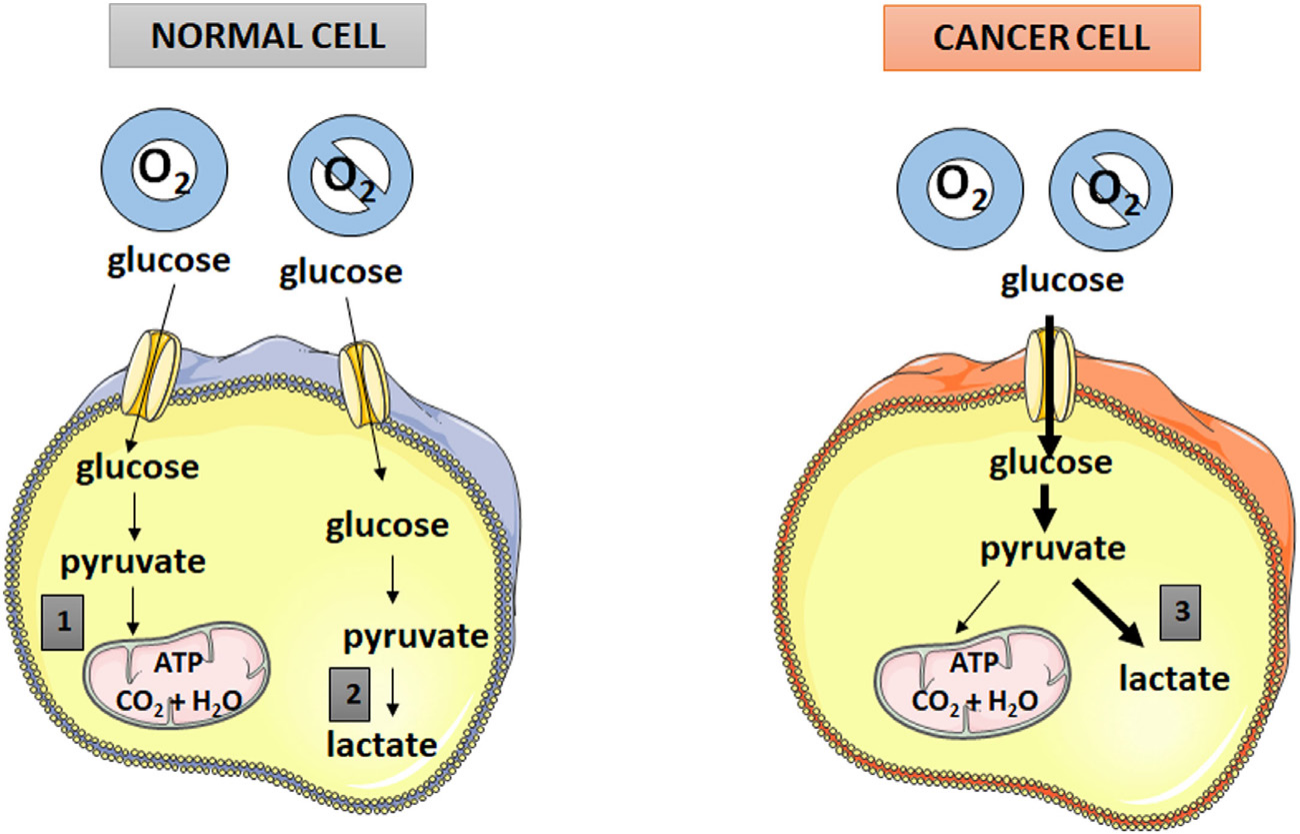
Comparison between glucose metabolism in normal and cancer cells. 1—oxidative phosphorylation. 2—anaerobic glycolysis. 3—aerobic glycolysis (aerobic lactatogenesis).

Cancer cell energy metabolism is an important hallmark of cancer (5) with deep interest as a molecular target for cancer therapy. Cancer cells, when compared with normal cells, are dependent in much higher rates of glucose uptake and, unlike normal cells, they exhibit an apparently energy inefficient metabolic switch in which they deviate glycolysis oxidation to lactate production even when oxygen is available (see “Glucose Uptake and Metabolism in Normal and Cancer Cells” for further details). This process, previously named “aerobic glycolysis” or the Warburg effect (5), but which we believe to match better an “aerobic lactatogenesis” (6), is believed to be a cancer cell fingerprint that can be used as a specific molecular therapeutic target (Figure 1). Therapeutic approaches directed toward this fingerprint will likely cause less adverse side effects thus contributing to reduce treatment-associated morbidities. Many compounds targeting energy metabolism are currently in trial or are approved as therapeutic agents against cancer (7). These include specific inhibitors of cancer-specific mutant isocitrate dehydrogenase (IDH 1 and/or IDH2), of the monocarboxylate transporter, critical for cancer cell nourishment using lactate, of the pyruvate dehydrogenase complex or of the mitochondrial complex I (7).

Current molecular targets for breast cancer therapy rely mainly on the expression of hormone receptors (estrogen and/or progesterone receptors) and of human epidermal growth factor receptor 2 (HER2). In each case, the expression of these receptors determines whether the patient should receive endocrine (hormone receptor-positive cases) and/or anti-HER2 antibody (HER2-positive cases) therapy (3).

Polyphenols, a heterogeneous family of natural compounds widely distributed in plants, are known to have a cancer-protective effect, which relies on their known antioxidant, anti-inflammatory, antiproliferative, pro-apoptotic, and antiangiogenic potential. In addition to this, many polyphenols have been shown to inhibit several steps in energy metabolism of cancer cells, namely glucose uptake and glucose metabolic enzymes. This paper intends to review the literature regarding the known antimetabolic effects of polyphenols, particularly in breast cancer cells.

## Polyphenols and Cancer

**Figure 2:**
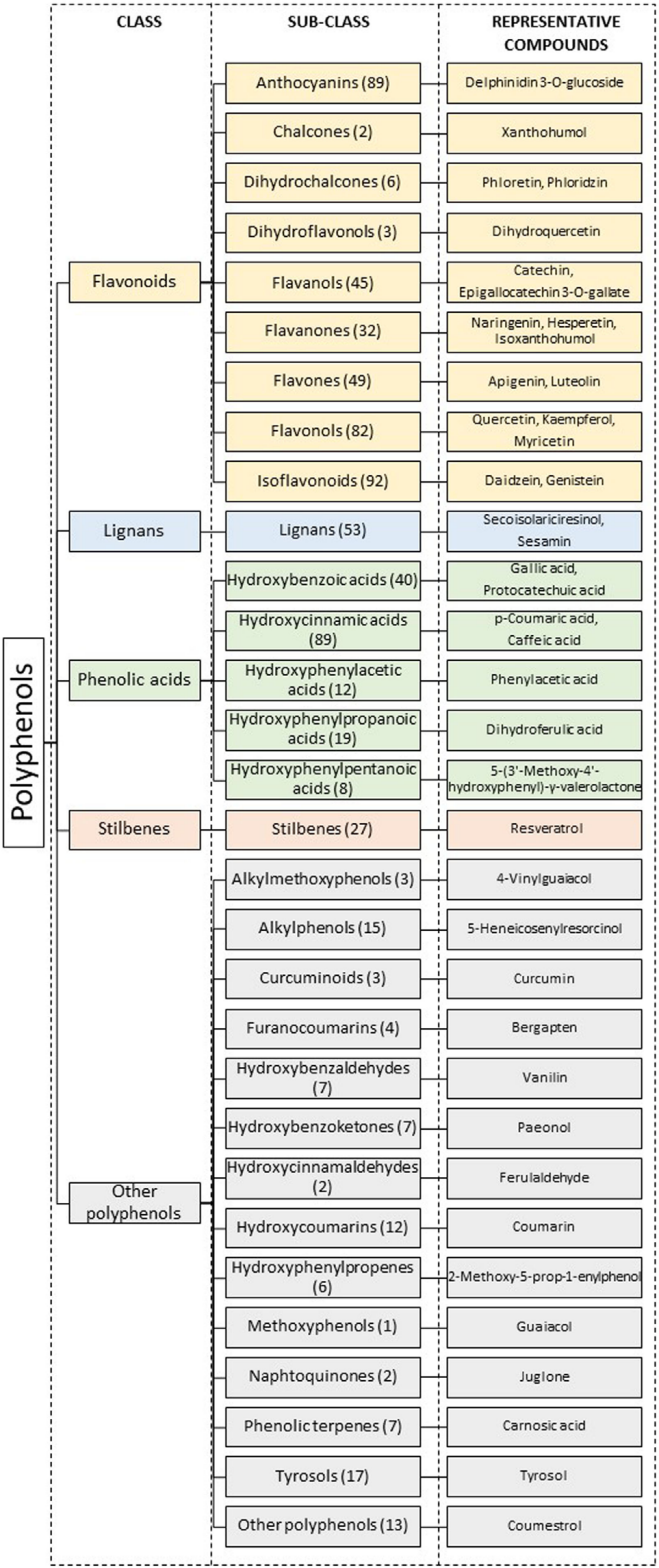
Classification of polyphenols according to their structures. Numbers in parenthesis represent the total number of compounds known in each sub-class. Adapted from Ref. (9–12).

Polyphenols, also called phenolic compounds, constitute a heterogeneous and large family of natural compounds widely distributed in plants. These compounds are products of plant secondary metabolism and act by protecting against stressors (e.g., UV light, pests) and in reproduction, by conferring colors to plants and consequently attracting insects for pollination (8). Polyphenols may be divided into classes and subclasses according to their chemical structures (Figure 2).

Polyphenols are an integral part of the human diet, and fruit and beverages such as tea and red wine represent the main sources of these compounds (13). For example, berry fruits are rich sources of anthocyanins, citrus fruits of flavanones, and apples of flavonols. Tea is a reach source of flavanols and coffee of hydroxycynnamic acids (8).

Research in polyphenols continues to increase, as demonstrated by the continuous increase in the number of papers regarding these compounds. A PubMed search with the term “polyphenols” shows that scientific production regarding this subject almost triplicated in the last decade (637 papers in 2007 up to 1,812 papers in 2017).

Much of the scientific interest in the study of polyphenols arises from studies showing that polyphenol-rich foods and beverages protect against chronic diseases including type 2 diabetes and cardiovascular diseases (8), obesity (4), and cancer (8, 14, 15).

Regarding their anticancer effect, polyphenols have shown bioactivity in several cancer molecular features, namely redox balance, cell proliferation, apoptosis, autophagy, angiogenesis, inflammation, expression of cell receptors or transcription factors, and synthesis of hormones. Recent reviews are available for detailed information (6, 14, 15) on these subjects. Molecular mechanisms involved in the anticancer effect of polyphenols in relation to breast cancer include interference with redox balance (acting either as antioxidant or pro-oxidant, they exert chemopreventive and antitumoral effects, respectively), cell cycle arrest, pro-apoptotic, autophagy activation, anti-inflammatory effect (inhibition of NF-kB, COX-2, and LOX), antiestrogenic effect, changes in ER expression, aromatase modulation, and interference with HER2 signaling (14).

Additionally, polyphenols may also interact with the microbiome: not only does gut microbiota transform many polyphenols, thus modulating their bioactivity, but also, polyphenols are known to modulate microbiome composition with a putative impact in human health (16). For example, Paul et al. have very recently gathered evidence that consumption of a genistein-enriched diet increased the abundance of members of *Lachnospiraceae* and *Ruminococcaceae* short-chain fatty acid (SCFA)-producing families, in microbiota of humanized mice (17). This was accompanied by a reduction in breast tumor size and an increase in breast tumor latency (period of time free of tumor, after injection of MDA-MB-231 breast cancer cells). The authors hypothesize that SCFA produced by the genistein-induced microbiota strains would epigenetically modulate tumor biology (17). Information on epigenetic regulation induced by gut microbiota can be found in a recently published review (18).

Also, polyphenols may exert cancer-protective effects directly through epigenetic modifications. Indeed, these dietary compounds have been shown to interfere with the three major epigenetic mechanisms, i.e., DNA methylation, histone modification, and non-coding RNAs, and these effects will also contribute to their anticancer potential (19–22), for example, in the restoration of the expression of tumor suppressor genes (6).

**Figure 3:**
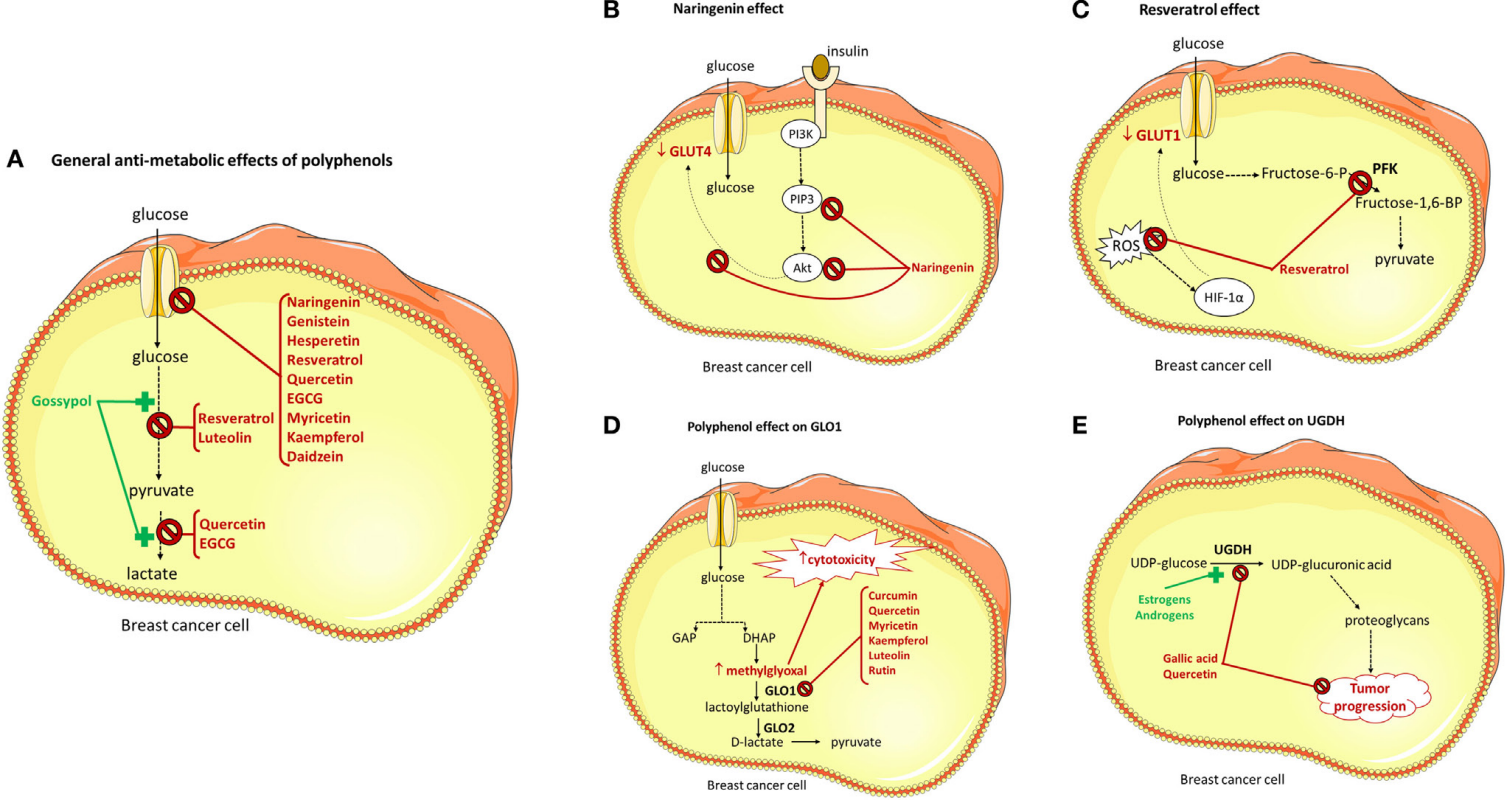
Effect of polyphenols on glucose cellular uptake and enzymes involved in glucose metabolism. GLUT, glucose transporter; PFK, phosphofructokinase-1; fructose-6-P, fructose-6-phosphate; fructose-1,6-BP, fructose-1,6-biphosphate; ROS, reactive oxygen species; GAP, glyceraldehyde-3-phosphate; DHAP, dihydroxyacetone phosphate; GLO1, glyoxalase-1; GLO2, glyoxalase-1; UGDH, UDP-glucose dehydrogenase; PI3K, phosphoinositide 3-kinase; PIP3, phosphatidylinositol 3,4,5-trisphosphate; Akt, protein kinase B; HIF-1α, hypoxia-inducible factor 1- α. **(A)** General antimetabolic effects of polyphenols, **(B)** naringenin effect, **(C)** resveratrol effect, **(D)** polyphenol effect on GLO1, and **(E)** polyphenol effect on UGDH.

Finally, it has becoming increasingly recognized that polyphenols may also interfere in glucose uptake and metabolism in cancer cells (Figure 3A). In this work, we will review the existing data showing that polyphenols act as metabolic antagonists for breast cancer cells.

## Glucose Uptake and Metabolism in Normal and Cancer Cells

Glucose is the primary energy source for many mammalian cells. This sugar can be either obtained from our diet or *de novo* synthesized in organs such as the liver and the kidney. Due to its low lipophilicity, transfer of glucose across biological membranes requires specific carrier proteins. In mammalian plasma membrane, two distinct families of transporters mediate glucose transfer: the sodium-dependent glucose co-transporters (SGLTs) and the facilitative glucose transporters (GLUTs).

The SGLT (gene symbol SLC5A) family of transporters are secondary active transporters that mediate glucose transport against its concentration gradient, coupled with sodium transport down its concentration gradient The Na^+^-electrochemical gradient is provided by the Na^+^–K^+^ ATPase pump (23). SGLT1, the first member of this family to be cloned, is a high-affinity glucose transporter found primarily in the apical membrane of enterocytes in the small intestine, with very small amounts detectable in the kidneys and the heart (22). SGLT2 is the major co-transporter involved in glucose reabsorption in the kidney, and SGLT2 inhibitors are a novel class of agents used to treat type 2 diabetes (24) (Table 1).

**Table 1:** The sodium-dependent glucose co-transporter (SGLT) and facilitative glucose transporter (GLUT) family of GLUTs.

Family	Isoform	Gene name	Tissue distribution	Substrate specificity
GLUT	GLUT1	SLC2A1	Ubiquotous (brain, red blood cells, colon, placenta)	Glucose/galactose
	GLUT2	SLC2A2	Intestine, liver, kidney, beta cells	Glucose/fructose/galactose
	GLUT3 (GLUT14)	SLC2A3	Brain, testis, kidney, placenta	Glucose/galactose
	GLUT4	SLC2A4	Skeletal and cardiac muscle cells, adipose cells	Glucose
	GLUT5	SLC2A5	Intestine, kidney, muscle, brain, testis	Fructose
	GLUT6	SLC2A6	Brain, spleen	Glucose
	GLUT7	SLC2A7	Small intestine, colon, testis, prostate, liver	Fructose, glucose
	GLUT8	SLC2A8	Testis, brain, fat, liver, spleen	Glucose/fructose
	GLUT9	SLC2A9	Kidney, liver, placenta, colon	Fructose/glucose
	GLUT10	SLC2A10	Heart, lung	Glucose
	GLUT11	SLC2A11	Muscle, heart, placenta, kidney, pancreas, fat	Glucose
	GLUT12	SLC2A12	Heart, prostate	Glucose/fructose

SGLT	SGLT1	SLC5A1	Intestine, trachea, kidney, heart, brain, testis, prostate	Glucose/galactose
	SGLT2	SLC5A2	Kidney, brain, liver, thyroid, muscle, heart	Glucose
	SGLT3	SLC5A4	Intestine, testis, uterus, lung, brain, thyroid	Glucose
	SGLT4	SLC5A9	Intestine, kidney, liver, brain, lung, trachea, uterus, pancreas	Glucose
	SGLT5	SLC5A10	Kidney	Glucose/galactose
	SGLT6	SLC5A11	Kidney, brain, intestine	Glucose

The GLUT (gene symbol SLC2A) family of facilitative transporters mediate glucose transport down its concentration gradient. This family of transporters includes fourteen members: GLUT1 to GLUT12, GLUT14 and the H^+^/myo-inositol transporter (HMIT) (21) (Table 1). GLUT1 is present in many tissues and is responsible for basal glucose uptake in most tissues (25). GLUT4, the insulin-responsive GLUT, is found in heart, skeletal muscle and adipose tissue, and constitutes the major cellular mechanism that diminishes blood glucose when carbohydrates are ingested (26). In healthy mammary glands, GLUT1 is the predominant isoform expressed, but GLUT8 expression was also reported (27).

Besides the ATP necessary to maintain normal cellular processes, highly proliferating tumor cells must also produce the extra ATP necessary to support three basic needs of these cells: maintenance of energy status, increased biosynthesis of macromolecules such as proteins, and maintenance of cellular redox status (28). It is now recognized that tumor cells reprogramme their metabolic pathways in order to meet this extra energetic demand necessary for tumor proliferation (28). One important metabolic change observed in tumor cells is the Warburg effect, which consists in a shift from ATP generation in the mitochondria through oxidative phosphorylation to cytosolic ATP generation through glycolysis with lactate production, even under normal oxygen concentrations (28, 29). So, distinctly from normal cells, many tumoral cells derive a considerable amount of their ATP by converting most incoming glucose to lactate (6) (Figure 1). This “aerobic glycolytic” (which we think should better be named “aerobic lactatogenic”) phenotype of tumor cells favors cell proliferation, and thus cancer progression, because (a) it generates high levels of glycolytic intermediates that contribute to biosynthetic pathways (5), and (b) it increases glucose metabolism, which appears to compensate for excess metabolic production of ROS in cancer cells (30). Aerobic glycolysis is now considered a key feature in cancer and has recently taken place in the famous ‘‘Hallmarks of cancer’’ described by Hanahan and Weinberg (5).

ATP production by “aerobic lactatogenesis” is far more rapid than ATP production by oxidative phosphorylation, but it is far less efficient. Indeed, up to 38 mol of ATP per mole of glucose is obtained when glycolysis is followed by the Krebs cycle and oxidative phosphorylation (31), but only 2 mol of ATP per mole of glucose is obtained when glycolysis is followed by lactate production, by oxidation of pyruvate mediated by lactate dehydrogenase (32). So, abnormally high rates of glucose uptake and oxidation must exist in cancer cells in order to support their increased energy, biosynthesis, and redox needs. Accordingly, cancer cells possess a 20- to 30-fold increased rate of glucose cellular uptake and a more than 30-fold higher glycolytic rate, when compared with normal cells (33). This increased dependence of cancer cells in relation to extracellular glucose levels, necessary to support high rates of glycolysis, makes interference with glucose cellular uptake, and glycolysis an attractive anticancer target (34–36).

Increased glucose cellular uptake in malignant cells has been associated with increased and deregulated expression of GLUT proteins (6). Overexpression of GLUT1 has been consistently observed in a great variety of cancers including breast, lung, renal cell, colorectal, and pancreas cancer (34, 37). GLUT1 is critical for glucose uptake in tumors (35, 38, 39), and is also the main transporter involved in glucose cellular uptake in several breast cancer cell lines (e.g., MCF-7 and MDA-MB-231) (40–42).

Besides GLUT1, other transporters of the GLUT family also appear to be involved in glucose cellular uptake by breast cancer cells in culture. More specifically, expression of GLUT3 and GLUT5 was also described (34, 37, 43). Moreover, expression of GLUT4 (42, 44–47) and insulin-stimulated glucose uptake were also found in some breast cancer cell lines (48–50). Although not much is known concerning the involvement of GLUT4 in cancer biology (51–53), downregulation of GLUT4 expression in the breast cancer cell lines MCF-7 and MDA-MB-231 cells impaired basal glucose uptake, promoted metabolic reprogramming from lactate production to oxidative phosphorylation and decreased cell proliferation and viability, strongly suggesting the participation of this transporter in basal glucose uptake in breast cancer cell lines with different degrees of malignancy and differentiation (54). Finally, GLUT12, which was proposed as a second insulin-responsive GLUT (55, 56), was also found to be expressed in MCF-7 cells (34, 57).

Glucose transporter-1 gene expression correlates with cancers of higher grade and proliferative index and lower degree of differentiation (35) and with increased malignant potential, invasiveness, and, consequently, poorer prognosis (41). GLUT1 has thus been proposed as an oncogene (34, 37, 38). Besides GLUT1, expression of other two GLUTs also present in breast cancer cells, namely GLUT3 and GLUT12 (see above), is also associated with a poor prognosis (34, 37). This demonstrates the extreme importance of glucose cellular uptake and GLUT transporters for tumor cell survival and proliferation.

Apart from GLUT family members, SGLT1 and SGLT2 overexpression has also been found in various cancers, such as pancreas, prostate, lung, liver, and lymph node cancer (58). However, SGLT expression levels in breast cancer cells have not been determined.

## Effect of Polyphenols on Glucose Transport in Breast Cancer Cells

Polyphenolic compounds are known to have anticancer effects, mediated by a plethora of cellular effects (see above). Interestingly, polyphenols have been found to interfere with both GLUT and SGLT transporters in several tissues. Consequently, they may interfere with glucose cellular uptake, as recently review in relation to glucose intestinal glucose absorption and placental glucose uptake (59). As mentioned above, the strict demand of cancer cells on glucose is dependent on an increased glucose uptake capacity, and for that reason inhibition of glucose cellular uptake may be a potential strategy for cancer therapy (34–36). However, the effect of polyphenols on glucose uptake by breast cancer cells and its relationship with the chemopreventive/anticarcinogenic effect of these compounds has been only scarcely studied (Table 2). These results were recently reviewed (6).

**Table 2:** *In vitro* effect of polyphenols and polyphenolic extracts on glucose uptake by breast cancer cell lines.

Compound	Concentration	Cell line	Effect	Mechanism of effect	Reference
Gossypol	10 µM	MCF-7	↑ in glucose consumption and lactate production ↔ in the ratio (lactate produced)/(glucose consumed)	*Quasi-competitive* inhibition	(60)

Naringenin	10 µM	MCF-7, T47D	↓ of basal and insulin-stimulated glucose uptake	Inhibition of MAPK-pathway	(44)

Genistein	10–100 µM	MCF-7, MDA-MB-231	↓ of glucose uptake	Not studied	(61)

Resveratrol	150 µM	T47D	↓ of glucose uptake	↓ GLUT 1 expression ↓ intracellular ROS causing ↓ of HIF-1α accumulation	(62)

Genistein, daidzein, and a soy seed extract	IC20 = 23, 52, and 166 µg/ml, respectively	MCF-7, MDA-MB-231	↓ of glucose uptake	Not studied	(63)

Hesperetin	50–100 µM	MDA-MB-231	↓ of basal and insulin-stimulated glucose uptake	↓ GLUT 1 expression ↓ cell membrane translocation of GLUT4	(48)

Quercetin, epigallocatechin-3-gallate	1–500 µM (26 min) 1–100 µM (4 h)	MCF-7, MDA-MB-231	↓ of glucose uptake	Competitive, independent of PKA, PKC, PKG, and calcium–calmodulin intracellular pathways	(45)

Myricetin, resveratrol genistein, kaempferol	100 µM 10–100 µM	MCF-7	↓ of glucose uptake	Mixed-type inhibition	(64)

*Petiveria alliacea* extract	3 µg/ml	4T1	↓ of glucose uptake	Not studied	(65)

Phloretin, quercetin	50–150 µM	HBL100	↓ of glucose uptake	Not studied	(66)

Catechin	100 µM (26 min)	MCF-7	↑ of glucose uptake (26 min)	Not studied	(64)

Cat:Lys complex	5 mM 1 mM	MCF-7	↑ of glucose uptake (26 min) ↓ of glucose uptake (24 h)	Not studied	(67)

Cat:Lys complex	5 mM 1 mM	MDA-MB-231	↑ of glucose uptake (26 min) ↑ of glucose uptake (24 h)	Not studied	(67)

*↑, increase; ↓, decrease; ↔ no effect*.

Gossypol (10 µM; 25 h), a polyphenolic bisnaphthalene aldehyde, markedly increased both glucose consumption, and lactate production in MCF-7 cells (Figure 3A). It should be noted, however, that no direct measurement of glucose uptake was made, and so the increase in glucose consumption may be the consequence of increased glycolytic rate or increased rate of glucose oxidation not related to glycolysis (e.g., pentose phosphate pathway). In this context, it is important to note that gossypol has been described to decrease glucose uptake in other cell types (60, 61, 68). Moreover, it is not apparent how the antiproliferative/cytotoxic effect of gossypol is associated with increased glucose consumption (6, 62). Finally, this effect was found with a non-physiological concentration of gossypol, as it is even higher than gossypol blood levels found in men taking gossypol as a contraceptive (0.2–0.4 µM) (69).

Naringenin (10 µM), a grapefruit flavanone, inhibited both basal and insulin-stimulated glucose uptake in two breast cancer cell lines (MCF-7 and T47D cells). The reduction in insulin-stimulated glucose uptake was verified in both proliferating and growth-arrested MCF-7 cells, and was not associated with changes in GLUT4 protein levels. Rather, naringenin was shown to inhibit stimulation of PIP3/Akt and p44/p42 MAPK activity, which were induced by insulin (49) (Figure 3B). Moreover, the antiproliferative effect of naringenin was mimicked by low-glucose conditions. So, it was concluded that naringenin inhibits proliferation of MCF-7 cells *via* impaired glucose uptake. Because physiologically attainable concentrations of naringenin reduced insulin-stimulated glucose uptake and showed an antiproliferative effect, the authors concluded that this compound possesses therapeutic potential as an anticancer agent (6, 49).

The flavonoid genistein (10–100 µM; 10 min), found in soybean, reduced glucose uptake in both estrogen receptor-positive MCF-7 and -negative (MDA-MB-231) breast cancer cell lines (70). These effects were observed with concentrations of genistein higher than the blood levels attainable with diet (even vegan diet) or even with genistein pill supplements in humans (64, 66).

Resveratrol (150 µM; 24 h), found in fruits such as grapes and berries, suppressed uptake of glucose and glycolysis in T47D breast cancer cells. Resveratrol was found to reduce GLUT1 expression. Moreover, its effect on glucose uptake was concluded to result from a reduction in intracellular ROS levels, which downregulates HIF-1α accumulation (63) (Figure 3C). As recently reviewed, the concentration of resveratrol used in this study is not achievable in humans, even when resveratrol pill supplements are used (67, 71), due to the low bioavailability of this compound resulting from extensive metabolism (6). However, the anticancer efficacy of resveratrol may be greatly increased by avoiding the oral route, as demonstrated by the observation that *in vivo* intraperitoneal injection of resveratrol (100 mg/kg) to mice with Lewis lung carcinoma was able to reduce fluorodeoxyglucose (^18^F-FDG) uptake by tumor cells (63).

The flavanone hesperetin (50–100 µM; 24 h), found in citrus fruits, decreased both basal and insulin-stimulated glucose uptake in MDA-MB-231 cells. Interestingly, the effect was distinct: the negative effect on basal glucose uptake resulted from GLUT1 downregulation, whereas the negative effect on insulin-induced glucose uptake was associated with impaired GLUT4 translocation to the cell membrane (46). Again, this inhibitory effect was found with hesperitin concentrations much higher than the blood concentrations observed in humans taking an hesperitin-rich (orange juice) diet (±0.05 μM) (65).

In another study, the flavonoids quercetin and epigallocatechin-3-gallate (EGCG) concentration-dependently inhibited glucose uptake by MCF-7 (10–23 µM; 26 min) and MDA-MB-231 (44–15 µM; 26 min) cells (50). This reduction in cellular glucose uptake was associated with a decrease in lactate production (Figure 3A). Quercetin and EGCG were found to be competitive inhibitors of glucose uptake in MCF-7 cells and their effect was independent of estrogen signaling and was not mediated by intracellular signaling pathways involving PKA, PKC, PKG, and calcium–calmodulin. Although not so potently, a longer (4 h) exposure to quercetin or EGCG caused also a decrease in glucose uptake, which was associated with an increase in GLUT1 mRNA expression. Additionally, both compounds presented an antiproliferative and cytotoxic effect in MCF-7 cells, which was more potent when glucose was present in the extracellular medium. So, quercetin and EGCG were concluded to inhibit basal glucose uptake and consequently lactate production in breast cancer cells and that these events are upstream determinants of their cytotoxic and antiproliferative effects (6, 50). The effective concentrations of quercetin and EGCG were higher than the blood levels found in humans, even after pill supplementation (72, 73).

The inhibitory effect of quercetin on glucose uptake by breast cancer cells was investigated in a later study. In this work, quercetin and phloretin were found to inhibit the growth of four distinct breast cancer cell lines (MCF-7, MDA-MB-231, HBL100, and BT549 cell lines). When investigated in more detail, it was concluded that both polyphenols (50–150 µM; 24 h) decrease glucose cellular uptake and lactate production in the HBL100 cell line, although no such effect was demonstrated in the MCF-7 cell line (74). The distinct effect of quercetin upon glucose uptake in MCF-7 cells between this work and the work by Moreira et al. (50) may be related to distinct times of exposure to this compound (4 vs 24 h). It should also be mentioned that the later work did not measure glucose uptake, but rather glucose remaining in the culture medium, and this parameter depends not only on glucose uptake but also on glucose metabolic rates.

A recent work showed that exposure to several polyphenols (myricetin, genistein, resveratrol, and kaempferol) reduced glucose uptake by MCF-7 cells (Figure 3A). Kaempferol was found to be the most potent inhibitor, with an IC_50_ of 4 µM. Kaempferol (30 µM) was concluded to inhibit GLUT1-mediated glucose uptake, as it decreased glucose uptake and downregulated GLUT1 mRNA expression. Interestingly enough, low-extracellular glucose mimicked the antiproliferative and cytotoxic properties of kaempferol, and high-extracellular glucose conditions prevented the effect of kaempferol. This clearly showed that inhibition of glucose cellular uptake mediates the anticancer effect of kaempferol in MCF-7 cells (6, 75). The concentrations found to be effective are higher than the blood levels of kaempferol attainable from diet in humans (76).

The effect of genistein, daidzein, and a soy seed extract on metabolomics of two distinct breast cancer cell lines was recently investigated (77). In MCF-7 cells, at relatively low concentrations (2–13 µM for genistein, 2–34 µM for daidzein, and 6–68 µg/ml for the soy seed extract), cell growth was stimulated, while higher concentrations had an inhibitory effect. In contrast, these compounds showed a concentration-dependent antiproliferative effect in MDA-MB-231 cells. Interestingly enough, the pro-proliferative effect of the compounds in MCF-7 cells was associated with an upregulation of the pentose phosphate pathway, and the antiproliferative effect of these isoflavones was associated with a significant decrease in glucose uptake. In contrast, in MDA-MB-231 cells, the antiproliferative effect was associated with inhibition of glutamine uptake and protein biosynthesis (77).

Several studies indicate that the anticarcinogenic efficacy of polyphenols can be enhanced by combining them with compounds such as amino acids and vitamins (78). In this context, a catechin:Lys complex (Cat:Lys) was recently tested in MCF-7 and MDA-MB-231 breast cancer cell lines. A short-term exposure (26 min) of both cell lines to Cat:Lys caused an increase in glucose uptake. Interestingly, a similar stimulatory effect of Cat in glucose uptake was observed in MCF-7 cells (75). However, when tested for a longer period (24 h), Cat:Lys decreases glucose uptake in MCF-7 cells and increases uptake in MDA-MB-231 cells (79). So, apparently, its antitumor effect is not related to the effect on glucose uptake, because Cat:Lys showed a similar antiproliferative, cytotoxic, antimigratory, and pro-apoptotic effect on both cell lines (75).

Finally, an extract of *Petiveria alliacea* (3 µg/ml; 48 h), known to contain flavonoids such as myricetin, was found to decrease glucose uptake and lactate production in the 4T1 breast cell line. However, no direct measurement of glucose uptake was done; rather, glucose levels in supernatant were measured (9).

So, for many of the investigated polyphenols, an inhibitory effect in relation to glucose uptake by breast cancer cells was found. Moreover, for some polyphenols, their anticancer effect was shown to be dependent on inhibition of glucose cellular uptake (6). The fact that, for kaempferol, inhibition of lactate cellular uptake was also demonstrated, raises the hypothesis that other polyphenolic compounds may also interfere with this mechanism. Compounds inhibiting both glucose and lactate uptake are very interesting in the context of cancer therapy, because they will deplete cancer cells of their two major energy substrates (6).

## Effect of Polyphenols on Glycolysis in Breast Cancer Cells

As mentioned above, cancer cells are dependent on high rates of glycolysis and lactate production. So, the enzymes contributing to glycolysis may represent an attractive target for cancer therapy. Nevertheless, only a few studies have described the ability of polyphenols to inhibit the glycolytic pathway in breast cancer cells, independently from inhibition of glucose cellular uptake (Table 3).

**Table 3:** *In vitro* effect of polyphenols on glycolysis in breast cancer cell lines.

Compound	Concentration	Cell line	Effect	Reference
Luteolin	50–100 µM (10 min)	4T1, MCF-7	↓ of glycolytic flux	(72)
Resveratrol	IC50 = 15 µM	MCF-7	↓ of PFK	(73)
1,2,3,4,6-penta-O-galloyl-β-d-glucose	40 µM (24 h)	MDA-MB-231	↓ of PC, ACYP2, ALDH3B1	(74)

*↑, increase; ↓, decrease*.

Luteolin (50–100 µM; 10 min), found in oregano and celery seed, was found to decrease the glycolytic flux in two distinct breast cancer cell lines, 4T1 and MCF-7 cells. Interestingly, enough, this effect was observed only under hypoxic conditions, and was not associated with inhibition of glucose cellular uptake. Although the concentrations of luteolin effective in reducing glycolytic flux were much higher than the diet-attainable concentration in humans (76), combination of luteolin (100 mg/kg) with doxorubicin had superior efficacy, and lesser toxicity compared with doxorubicin alone, in relation to decrease in tumor size and weight loss, in mice. So, it was concluded that luteolin, as a glycolytic inhibitor, might be a new adjuvant agent for chemotherapy (10).

6-Phosphofructo-1-kinase-1 (PFK) is a critical glycolytic enzyme, and its activity is directly correlated with cellular glucose consumption (11). Gomez et al. showed that resveratrol (1–100 µM; 24 h) causes a decrease in cell viability, glucose consumption, and ATP content in MCF-7 cells, and that these effects are correlated with PFK inhibition. Furthermore, they showed that resveratrol directly inhibits PFK (Figure 3C), causing a decrease in both affinity and V_max_, by promoting the dissociation of the enzyme from fully active tetramers into less active dimers and that its effect is exacerbated by known negative regulators of the enzyme (such as ATP and citrate) and prevented by positive modulators (such as fructose-2,6-bisphosphate and ADP) (11). In summary, resveratrol was concluded to cause direct inhibition of PFK activity, therefore disrupting glucose metabolism and reducing breast cancer cell viability (11). Of relevance, the lowest concentration of resveratrol found to be effective (1 µM) is attainable in human blood after pill supplementation (71).

Besides glycolytic enzymes, some polyphenols were found to interfere with other enzymes participating in glucose utilization by cancer cells, as shown next.

1,2,3,4,6-penta-O-galloyl-beta-d-glucose (PGG), a polyphenolic compound isolated from *Rhus chinensis*, is known to have antitumor, antiangiogenic, and antidiabetic activities. Recently, this compound was shown (by microarray data and real-time RT-PCR) to cause a significant downregulation of genes involved in pyruvate metabolism, namely pyruvate carboxylase, acylphosphatase, and ALDH3B1 (aldehyde dehydrogenase). These findings led the authors to suggest the potential of PGG as anticancer agent for breast cancer cells by targeting cancer metabolism genes (12).

Glyoxalase-1 (GLO1) is a ubiquitous cellular enzyme that participates in the detoxification of methylglyoxal (MG), a cytotoxic by-product of glycolysis (80). The expression of GLO1 is correlated to the flux of glucose being oxidized in the glycolytic pathway, and because cancer cells must increase their glycolytic flux several fold in order to obtain the necessary amount of ATP (see above), this increases the level of MG to toxic concentrations. Consequently, most cancer cells show increased expression of GLO1 (80). Interestingly enough, several polyphenols (curcumin, quercetin, myricetin, kaempferol, luteolin, and rutin) were found to inhibit GLO1 specific activity (Figure 3D). Of these, curcumin was found to be the most potent (Ki = 5 μM). The authors presented evidence that inhibition of GLO1 by curcumin may result in non-tolerable levels of MGO and GSH, which, in turn, may lead to depletion of cellular ATP and GSH, accounting to the antitumor efficacy of curcumin in several cell lines, including two breast cancer cell lines (JIMT-1 and MDA-MB-231) (81). Of note, curcumin blood levels in humans are lower than 1 µM, even after curcumin pill supplementation (82).

Uridine diphosphate (UDP)-glucose dehydrogenase (UGDH) catalyzes the oxidation of UDP-glucose to yield UDP-glucuronic acid, a precursor for the biosynthesis of glycosaminoglycans and proteoglycans (83). Increases in UDP-glucuronic acid levels can cause excessive production of proteoglycans, compounds that have been implicated in the progression of breast cancers (83). Moreover, upregulation of UGDH by estrogen and androgens is known to be present in estrogen-responsive breast cancer cells (84). In this context, gallic acid and quercetin (300 µM; 24 h) were found to decrease the specific activity of UGDH at the post-translational level (Figure 3E). Gallic acid appears to be a non-competitive inhibitor of the enzyme, whereas quercetin appears to be a competitive inhibitor. Because these two compounds inhibited the proliferation of MCF-7 human breast cancer cells, it was concluded that gallic acid and quercetin are effective inhibitors of UGDH that exert strong antiproliferative activity in breast cancer cells (85).

## Conclusion and Future Perspectives

Despite the high-survival rate in breast cancer patients and the availability of well-designed and effective therapeutic strategies, especially for hormone receptor or HER2-positive breast cancer, there is still the need for more drug research particularly regarding triple-negative breast cancer, because of its unresponsiveness to hormone or anti-HER2 therapy. In fact, drug discovery lies in the list of cancer research priorities in the United States set by the Lancet Oncology Commission in the end of 2017 (4).

Cancer cell energy metabolism is an important target for improved therapeutic strategies. In this regard, polyphenols may add an important contribution for anticancer therapy. Indeed, many of these phytochemicals have been shown to regulate redox balance, cell proliferation, apoptosis, autophagy, angiogenesis, inflammation, cell receptor and transcription factor expression, hormone synthesis, microbiota composition, and epigenetic mechanisms, all of which may underlie carcinogenesis and tumor progression. In addition, as exposed in this review, polyphenols from almost all classes (Figure 2) have been described to target glucose uptake or metabolism in breast cancer cells.

The studied mechanisms underlying glucose uptake inhibition in breast cancer cells are also diverse, ranging from direct functional effect upon the transporter, inhibition of transporter gene expression, impairment of membrane translocation processes, and redox balance modulation with implications in the accumulation of a transcription factor (Table 2). The effect of polyphenols upon glycolysis is less studied, but seems to involve direct inhibition of glycolytic enzymes and inhibition of enzyme gene expression.

An important point concerns the fact that, for most of the polyphenols presented above, their inhibitory effect upon glucose transport or metabolism was found, *in vitro*, with higher than physiological human blood concentrations. So, the *in vivo* efficacy of these compounds in inhibiting glucose transport and metabolism, as contributing to their antitumor effect, should be addressed.

To date, clinical trials in cancer patients using polyphenols to ensure their safety and/or efficacy are still largely lacking. Nevertheless, some studies have been conducted; some of them had disappointing results, but some deserve further studies. For example, clinical studies using gossypol have shown that this polyphenol seems to be safe; however, it presented a negligible antitumor effect in advanced breast cancer (86). Moreover, although soy isoflavone supplementation have proven to be uneffective in breast cancer prevention (87), a phase II placebo-controlled, randomized, double-blind clinical trial in prostate cancer patients evidenced genistein as a well-tolerated chemopreventive agent (88, 89). To our knowledge, no trials have been performed with genistein in breast cancer patients, and this issue deserves further attention. Finally, EGCG has been recently tested in a phase II randomized, double-blind, presurgical trial with bladder cancer patients. ECGC was detected in plasma, urine, and bladder tissue in a dose-dependent profile, after administration of a green tea polyphenol formulation rich in this polyphenol, and it was found to modulate tissue biomarkers of proliferation and apoptosis (86). This trial indicates that ECGC could be a promising natural compound for a clinical trial in cancer patients, including breast cancer. This same formulation rich in EGCG has been found to be well-tolerated in another trial with patients with Barrett’s esophagus (90).

Another important point relates to the fact that the effect of polyphenols in normal breast cells has not been addressed. So, it is reasonable to question whether it is appropriate to non-selectively target molecules expressed both in normal and in cancer cells. However, we know that cancer cells are likely to suffer more severely than normal cells from glucose deprivation, given their extremely higher dependence on high amounts of glucose. In this context, it should also be considered that compounds targeting upstream players in glucose metabolism (GLUTs or enzymes in the first step of glycolysis) are likely to induce more adverse side effects, given that this inhibition will be less glycolysis-specific, when compared with compounds targeting downstream players. One example of this is the failure of 2-DG, an inhibitor of GLUT1, as a chemotherapeutic agent (7). So, research on the effects of polyphenols on glucose metabolism of (breast) cancer cells should be fostered in the near future.

## Author Contributions

EK and FM equally contributed to the manuscript conception and writing.

## Conflict of Interest Statement

The authors declare that no competing financial interests exist and that they have no conflict of interest.
